# Supplementary material for: WordCommentsAnalyzer: A windows software tool for qualitative research
Source: F1000Res. 2018 Sep 4;7:536. Originally published 2018 May 3. [Version 2] doi: 10.12688/f1000research.14819.2 (PMC6137411; doi:10.12688/f1000research.14819.2)
Supplement: Supplementary file 3 [file f1000research-7-17614-s0002.tgz › b2a79496-4ada-47c4-9682-a99ce867836d.zip!/Eight Iranian Health Journals/Journal of Knowledge and Health_39.docx]

© Journal of Knowledge and Health. This work is licensed under CC BY 4.0 (https://creativecommons.org/licenses/by/4.0/).

Volume & Issue: دوره 3، شماره 2: 1387

Title: عوامل جدید پیش¬گویی¬کننده خطر (پیش¬آگهی) برای گانگرن فورنیه؛ تجربه 10 ساله

http://knh.shmu.ac.ir/index.php/site/article/view/205

مقدمه: ما در این مطالعه عوامل خطر و پیش­گویی کننده گانگرن فورنیه را مورد بررسی و ارزیابی قرار داده­ایم. مواد و روش­ها: در این تحقیق علایم بالینی، ارزيابی شاخص­های آزمایشگاهی (آلبومین، کراتینین، اوره، سدیم، پتاسیم، کلسیم، گلوبول­های سفید و هماتوکریت) و عوامل خطر احتمالی تعداد 71 بیمار مراجعه­کننده به مرکز آموزشی درمانی قائم مشهد (1387-1377) با تشخیص گانگرن فورنیه تحت بررسی قرار گرفته است. نتايج: ميانگين سنی بیماران 0/17 ± 8/60 سال و فاصله زمانی بین شروع علایم تا مراجعه به مرکز درمانی 2/5 ± 8/6 روز گزارش شده است. منشاء عفونت در بیماران، 35% پوستی، 48% ادراری و 17% رکتال بود. 16 نفر از بیماران فوت­نموده و تفاوت معناداری بین میانگین درجه حرارت در زمان ورود، ضربان قلب و تعداد تنفس در دو گروه فوت­شده و بهبودیافته مشاهده نشد. در مقایسه بین دو گروه تفاوت معناداری برایWBC ،Na ، K،Cr  یافت نشد. اما در ارزیابی اوره، آلبومین، کلسیم و سطح درگیری عفونت این تفاوت­ها معنادار بود. نتیجه­گیری: در مقایسه با شاخص شدت گانگرن فورنیه لائور، شاخص­های درجه حرارت، ضربان قلب، تعداد تنفس، سطح سدیم و پتاسیم سرم، سطح کرانینین، هماتوکریت و WBC دارای ارزش پیش­گویی­کننده بین دو گروه بهبود یافته و فوت­شده نبودند و فاصله زمانی شروع علایم تا مراجعه به پزشک و شروع درمان، سن بیماران، منشاء عفونت (پیش­آگهی بد برای منشاء رکتال)، سطح درگیری عفونت، سطح آلبومین، اوره، کلسیم سرم و تعداد موارد دبریدمان به­عنوان عوامل پیش­گویی­کننده مطرح می­باشند.

Title: بررسي رابطه کفایت دياليز با دز اريتروپوئیتين مكمل تجويزشده در بيماران همودياليزی

http://knh.shmu.ac.ir/index.php/site/article/view/208

مقدمه: اين تحقيق با هدف تعيين رابطه كفايت دياليز با دز اريتروپوئيتين مكمل تجويز شده در بيماران تحت درمان با همودياليز شهرستان شاهرود انجام شده است. مواد و روش­ها: نمونه­هاي پژوهش شامل 54 بيمار تحت درمان با همودياليز در شهرستان شاهرود بودند. معیار ورود نمونه شامل درمان با همودیالیز به­مدت 6 ماه، درمان با اریتروپوئیتین زیر جلدی حداقل برای 4 ماه، فریتین پلاسمای بیش­تر از 100 نانوگرم در میلی­لیتر و هماتوکریت هدف 30% برای مدت 3 ماه بود. برای بررسی کفایت دیالیز انجام شده ضریب Kt/V برای بیماران محاسبه شده است. نتایج: در این مطالعه 30 نفر (6/55%) بيماران مذكر و 24 نفر (4/44%) مؤنث بودند. 4/70% بيماران (38 نفر) داراي ضريب Kt/V كم­تر از 2/1 و 6/29% (16 نفر) داراي ضريب Kt/V بيش­تر از 2/1 بودند. آزمون آماري t مستقل نشان­دهنده عدم وجود اختلاف معنادار بين ميزان آهن، فريتين، هموگلوبين و هماتوكريت در بيماران با Kt/V كم­تر و بيش­تر از 2/1 بود، اما بين ميزان تزريق اريتروپوئيتين و ضريب Kt/V ارتباط معناداري مشاهده گرديد، به­طوري­كه ميانگين تزريق ماهانه اريتروپوئيتين در بيماران با 2/1<Kt/V كم­تر از ميانگين آن در بيماران با 2/1>Kt/V بود. نتيجه­گيري: يافته­هاي حاصل از اين پژوهش و ساير پژوهش­ها در زمينه تجويز اريتروپوئیتين در بيماران مبتلا به نارسايي مزمن كليه تحت درمان با دياليز مبين آن است كه انجام دياليز كافي (Kt/V بيش­تر از 2/1) مي­تواند ضمن كاهش نياز بيماران به اريتروپوئيتين مكمل و هزينه­هاي ناشي از آن، سبب كاهش عوارض احتمالي اين دارو نيز گردد.

Title: بررسی ارتباط غلظت منوکسیدکربن در هواي شهر تهران با فشارخون بارداری و پره¬اکلامپسیا

http://knh.shmu.ac.ir/index.php/site/article/view/209

مقدمه: در این مطالعه ارتباط آلودگي هواي مناطق شهري تهران از نظر غلظت گاز منوکسیدکربن با فشارخون بارداری و پره­اكلامپسيا در مادران ساكن نواحی مختلف شهر با سطوح مختلف آلودگی، مورد بررسی قرار گرفته است. مواد و روش­ها: در اين مطالعه 4500 خانم باردار از سه بيمارستان وابسته به دانشگاه تهران كه داراي زايشگاه بودند انتخاب شدند. این مادران در مناطق با آلودگي پایین CO (ایستگاه شرق) و سه ایستگاه با آلودگي متوسط تا شديد CO (ایستگاه مرکزی، جنوب و غرب تهران)زندگی مي­کردند. فاصله محل زندگی نمونه­ها از ایستگاه­هاي سنجش آلودگي حداکثر 5 کيلومتر بوده است.  ابزار جمع­آوري داده­ها شامل فرم جمع­آوري اطلاعات دموگرافیک و اطلاعات بارداری بود. تشخیص بيماري فشارخون بارداری و پره­اکلامپسیا توسط متخصصین زنان (همكاران طرح) در بیمارستان­های مورد پژوهش انجام پذيرفت. فشارخون دیاستولیک و سیستولیک 6 ساعت پس از زایمان برای کلیه­ی مادران در شرایط استاندارد ثبت گردید. نتایج: در مناطق با آلودگي بالاي CO نسبت به مناطق با آلودگي پايين، ميانگين فشار خون دياستولي در نمونه­هاي پژوهش بیشتر و ميانگين فشار خون سيستولي بطور معناداري کمتر گزارش شده است. بروز فشارخون بارداری و پره­اکلامپسیا در مناطق با آلودگی هوای مختلف تفاوت معناداری را نشان نمی­دهد. نتیجه­گیری: نتایج این مطالعه نشان داد که در معرض بودن با آلودگی محیطی به CO می­تواند عاملی مؤثر در کاهش یا افزایش فشارخون بارداری باشد. با توجه به اینکه علت و مکانیسم اصلی فشارخون ناشی از حاملگی در بسیاری از موارد روشن نیست و هم­چنین از سوی دیگر تأثیر سطوح پایین CO روی فشارخون بارداری به­خوبی آشکار نشده است، لذا مطالعات بیشتری توصیه می­شود.

Title: اجراي شكل تغییریافته يادگيري براساس حل مسأله (MPBL) و بررسي نظرات دانشجويان

http://knh.shmu.ac.ir/index.php/site/article/view/211

مقدمه: استفاده از یادگیری بر اساس حل مسأله (Problem based learning) در سرتاسر دنيا گسترش يافته است و از اين استراتژي آموزشي در بسياري از دانشگاه­هاي دنيا به صور مختلف سود برده مي­شود. به­دليل مشكلات مربوط به اجراي روش يادگيري بر اساس حل مشکل پژوهش­گران با توجه به محدودیت­های موجود اقدام به تغییر شکل مرسوم يادگيري بر اساس حل مشكل نمودند. مواد و روش­ها: در اين پژوهش در درس پرستاري بيماري­هاي داخلي جراحي 4 به ميزان 5/1 واحد، روش اصلاح شده يادگيري بر اساس حل مسأله (MPBL) طراحي و اجرا شد. تعداد 31 دانشجويي كه اين درس را انتخاب كرده بودند به­عنوان واحدهاي پژوهش در نظر گرفته شدند. در پايان هر موضوع درسي، از دانشجويان خواسته مي­شد تا نظرات خود را در مورد اين روش در برگ پرسش­نامه منعكس نمايند. نتايج: از داده­هاي به­دست آمده در اين پژوهش مشخص گرديد كه در روش اصلاح شده يادگيري بر اساس حل مسأله، بخش­هايي از درس كه بيش­تر جنبه باليني و كاربردي داشتند از نظر دانشجويان با انگيزه و تمايل دروني بيش­تري همراه بوده است. علاوه بر اين دانشجويان به­طور كلي (4/77%) از روش اجرا شده رضايت داشتند. نتیجه­گیری: بر اساس تجربه اين تحقيق مزاياي روش MPBL نسبت به PBL آن است كه: 1- مدرس در روش PBL فعاليت اندكي دارد كه از معايب اين روش است اما در MPBL فعاليت معلم به مراتب بيش­تر است و احساس مؤثر بودن بيش­تري مي­نمايد. 2- نياز به بكارگيري تعداد زيادي مدرس براي اداره گروه­ها نيست. 3- تعداد بيش­تري دانشجو را مي­توان در مدت كوتاه­تري آموزش داد. 4- بار مالي قابل توجهي به سيستم تحميل نمي­كند.

Title: بررسی مقایسه‌ای وضعیت شناختی و افسردگی سالمندان مقیم سرای سالمندان و ساکن منازل شخصی

http://knh.shmu.ac.ir/index.php/site/article/view/212

مقدمه: در این پژوهش وضعیت شناختی و افسردگی سالمندان مقیم خانه سالمندان و ساکن منازل شهر شاهرود در سال 86 ارزیابی و مقایسه شده است. مواد و روش­ها: این مطالعه بر روی 46 سالمند مقیم خانه سالمندان و 90 نفر از سالمندان ساکن در منازل شخصی انجام شده است. پرسش­نامه­های استاندارد بررسی وضعیت شناختی (Minimal Mental Status Examination) و فرم کوتاه بررسی افسردگی در سالمندان (Geriatric Depression Scale) پس از معاینه برای دو گروه تکمیل گردید. نتایج: در خصوص بررسی وضعیت شناختی در سالمندان مقیم سرای سالمندان، 13 نفر (3/28%) اختلال شناختی خفیف (21 ≤ MMSE)، 16 نفر (8/34%) اختلال شناختی متوسط (21 > MMSE ≥10) و 17 نفر (37%) اختلال شناختی شدید (10 > MMSE) داشتند، در حالی­که در سالمندان ساکن منازل شخصی اختلال شناختی خفیف، متوسط و شدید به­ترتیب در 49، 40 و 1 نفر گزارش شده است. در خصوص بررسی حالت افسردگی، در سالمندان مقیم سرای سالمندان 14 نفر (4/30%) وضعیت طبیعی، 12 نفر (1/26%) افسردگی متوسط (10≥ GDS > 3) و 20 نفر (5/43%) افسردگی شدید (10 < GDS) داشتند، در گروه دوم 51 نفر (56%) وضعیت طبیعی، 29 نفر (9/31%) افسردگی متوسط و 10 نفر (11%) افسردگی شدید داشتند که اختلاف بین دوگروه معنادار می­باشد. نتیجه­گیری: اختلال شناختی و شدت افسردگی در سالمندان مقیم خانه سالمندان به­طور معناداری بیش­تر گزارش شده، که با تقویت ارتباطات خانوادگی و برنامه­های ورزشی می­توان آن­ها را کاهش دهیم.

Title: بررسي ده ساله علل مرگ¬ومير مادران باردار در استان کهگيلويه و بويراحمد

http://knh.shmu.ac.ir/index.php/site/article/view/213

مقدمه: شاخص ميزان مرگ­ومير مادران نشان­دهندۀ وضعيت کلي سلامت زنان در جامعه است. اين مطالعه به­منظور تعيين وضعيت مرگ­ومير مادران باردار در یکی از استان­های کم­تر توسعه یافته صورت گرفته است. مواد و روش­ها: اين مطالعه به­صورت مقطعي و توصيفي تحليلي و با استفاده از داده­هاي موجود در سيستم شبکه­اي بهداشت و درمان استان در فاصله سال­هاي 1376 تا 1386 انجام شده است. هم­چنين دسترسي به بخشي از اطلاعات مورد نياز از طريق اداره سلامت مادران حوزۀ معاونت سلامت وزارت بهداشت تأمين گرديده است. نتایج: بر اساس يافته­هاي اين تحقيق در طي دوره­ی ده ساله­ی 1376 تا 1386 به­طور متوسط ميزان مرگ مادر باردار 7/57 در صدهزار تولد زنده (66 مورد) بوده است. بيش­ترين موارد مرگ مادر در سال 1379 (13 مورد) و کم­ترين آن در سال 1383 (1 مورد) گزارش شده است. خونريزي به­عنوان شايع­ترين علت مرگ به تنهايي 41 درصد (27 مورد) علل مرگ را به خود اختصاص داده است. نتيجه­گيري: براساس يافته­هاي اين تحقيق پايين بودن سطح سواد مادر، سابقۀ حاملگي بيش از 4 بار، سکونت در روستا (بالاخص روستاي قمر)، عدم دريافت مراقبت­هاي ويژۀ دوران بارداري و عدم دسترسي به خدمات فوريت­هاي مامايي از مهم­ترين عوامل مؤثر بر مرگ مادران در استان کهگيلويه و بويراحمد بوده­اند.

Title: ارتباط افزايش وزن مادر در دوران بارداري با وزن هنگام تولد نوزاد

http://knh.shmu.ac.ir/index.php/site/article/view/214

مقدمه: وزن هنگام تولد يكي از عوامل اصلي تعيين­كننده رشد جسماني و مغزي كودك بوده و نشانه معتبري از رشد داخل رحمي مي­باشد. اين پژوهش به­منظور تعيين ارتباط افزايش وزن مادر در بارداري با وزن هنگام تولد نوزاد، انجام شده است. مواد و روش­ها: تعداد 600 زن حامله از بین مراجعین به بیمارستان هاجر شهرکرد پس از کسب رضایت انتخاب شدند. ابزار گرد­آوری اطلاعات در این پژوهش پرسش­نامه بود و تکمیل آن بر­اساس مصاحبه با مادران، مطالعه پرونده دوران بارداري، پرونده زايمان، پرونده نوزاد و توزین مادران و نوزادان با استفاده از وزنه­های یکسان بوده است. سن كم­تر از 18 و بيش­تر از 35 سال مادر، سن حاملگي كم­تر از37 هفته، حاملگي چند قلو، سابقه سقط و مرگ داخل رحمي جنين، استعمال سيگار و الكل و ابتلا به هر نوع بیماری زمینه­ای در مادر و عدم رضایت شرکت در مطالعه معیار خروج فرد از مطالعه قرار گرفته است. نتايج: ميانگين افزايش وزن مادر در دوران بارداري 6/3 ± 5/11 كيلوگرم بود اين میزان با افزايش تعداد زايمان (015/0P=) و با افزايش ميزان تحصيلات مادر (018/0P=) افزايش­يافته و هم­چنين در زايمان به­روش سزارین بيش­تر از زايمان به­روش واژینال بود (006/0P=). ميانگين وزن هنگام تولد نوزادان 1/433 ± 1/3179 گرم گزارش شد که در زنان چندزا بیش­تر از اول­زا (003/0P=)، در نوزادان پسر بیش­تر از نوزادان دختر (013/0P=) و در زنان تحصیل­کرده بیش­تر از زنانی بود که تحصیلات پایین­تری داشتند. هم­چنین میانگین وزن هنگام تولد نوزاد با افزایش وزن مادر چه در شروع بارداری و چه در طول دوران بارداری، افزایش یافته است. نتيجه­گيري: نتايج اين پژوهش اهميت وزن مادر چه در شروع بارداري و چه در طول بارداري را به­عنوان شاخصي براي شناسايي مادراني قرار مي­دهد كه در معرض خطر تولد نوزاد كم وزن مي­باشند. متعاقب شناسايي اين مادران، مي­توان از آن­ها مراقبت بيش­تري به­عمل آورد و آموزش­هاي لازم را در زمينه­هاي مختلف به آنان داد.

Title: بررسی ميزان آلومينيوم در چای مصرفی کشور

http://knh.shmu.ac.ir/index.php/site/article/view/215

مقدمه: برگ سیاه چای یکی از منابع مهم آلومینیوم در رژیم غذایی است. اين مطالعه با هدف بررسی ميزان آلومينيوم در اينفوزيون چای مصرفی کشور طراحی و اجرا شده است. مواد و روش­ها: به­منظور پایش آلومینیوم در چای مصرفی ایرانی 27 نوع چاي (نمونه­های داخلی و خارجی) از سوپرمارکت­ها و مغازه­هاي سطح شهر خريداري شد و پس از تهيه اينفوزيون در زمان­های 10 و 60 دقيقه غلظت آلومينيوم توسط روش جذب اتمی قرائت شد و نتايج به­دست آمده توسط نرم­افزار  SPSS مورد تجزيه و تحليل قرار گرفت. نتایج: در 10 دقيقه اينفوزيون چای باروتی با 59/1 حداقل و بامداد با 60/18 ميلی­گرم بر ليتر حداکثر مقدار آلومينيوم را نشان دادند. هم­چنين در 60 دقيقه اينفوزيون چای ترش مکه با 28/1 حداقل و گلستان با 2/27 ميلی­گرم بر ليتر حداکثر مقدار آلومينيوم را نشان دادند. آناليز آماری نتايج با استفاده از آزمون t زوج نشان می­دهد که زمان اينفوزيون به­طور معناداری بر ميزان نشت آلومينيوم به داخل اينفوزيون مؤثر نبوده و با افزايش زمان اينفوزيون غلظت آلومينيوم افزايش نمی­يابد (05/0<P). محاسبات نشان دادند که اگرچه 1 ليتر چای 68/17 درصد دريافت روزانه خوراکی آلومينيوم را تشکيل می­دهد، درصد در دسترس برای جذب در دستگاه گوارش فقط 49/8 درصد متوسط غلظت کل آلومينيوم می­باشد. نتیجه­گیری: بنابراين بر اساس يافته­های این مطالعه چنين می­توان گفت که نوشيدن چای به مقدار متوسط نمی­تواند هيچ اثر سمی بر انسان داشته باشد. اگرچه ذکر اين نکته ضروری است که نوشيدن چای ممکن است در افرادی که جذب يا دفع آلومينيوم در بدن آن­ها دچار مشکل است سميت آلومينيوم ايجاد کند.
